# Increased Frequency of Bone Marrow T Follicular Helper Cells in Patients with Immune-Related Pancytopenia

**DOI:** 10.1155/2013/730450

**Published:** 2013-08-27

**Authors:** Hong Yu, Jiangbo Zhang, Rong Fu, Hui Liu, Huaquan Wang, Kai Ding, Yihao Wang, Lijuan Li, Honglei Wang, Zonghong Shao

**Affiliations:** Department of Hematology, Tianjin Medical University General Hospital, 154 Anshan Street, Heping District, Tianjin 300052, China

## Abstract

Immune-related pancytopenia (IRP) is one kind of bone marrow failure diseases which is related to autoantibodies. Autoantibodies have been detected on the membrane of various bone marrow (BM) hemopoietic cells by BM mononuclear-cell-Coombs test or flow cytometric analysis. There are autoantibodies in the BM supernatant of IRP patients, which can target several antigens on hematopoietic cells membranes by western blot. T follicular helper (Tfh) cells are the true helper cells for Ab responses, which represent one of the most numerous and important subsets of effector T cells. Dysregulation of Tfh cell function or expression of Tfh cell-associated molecules could contribute to the pathogenesis of autoimmune diseases. Currently, there are no studies regarding the role of Tfh cells in IRP patients. The percentages of Tfh cells, Tfh-related molecules ICOS, CD40L, IL-21, and Bcl-6 in BM were investigated in 90 patients with IRP, and 25 healthy controls. We observed that there exist increased quantity and hyperfunction of Tfh cells in IRP, and the results were correlated with patient characteristics. It was indicated that dysregulated Tfh cells might be involved in the pathogenesis of IRP and that inhibition of Tfh cells effector molecules might provide opportunities for new therapeutic approaches to IRP and even other human autoimmune diseases.

## 1. Introduction

Over the last decade, we have described a group of patients with hemocytopenia who did not conform to the diagnostic criteria of known hematological and nonhematological diseases, such as aplastic anemia (AA), myelodysplastic syndrome (MDS), paroxysmal nocturnal hemoglobinuria (PNH), megaloblastic anemia (MA), iron deficiency anemia (IDA), anemia of chronic disease (ACD), autoimmune hemolytic anemia (AIHA), and congenital anemia. Most patients had a good response to adrenocortical hormone (ACH) and/or high-dose intravenous immunoglobulin (IVIG) treatment, which indicated that the cytopenia might be mediated by autoantibodies [1–3]. We detected autoantibodies on the membrane of BM hemopoietic cells by bone marrow mononuclear-cell-(BMMNC-) Coombs test [4–6] or flow cytometric analysis. The positive rate was 67% and 86%, respectively [7], indicating that this was an autoimmune disease. We termed this abnormality “immune-related Pancytopenia” (IRP) (also known as “cytopenia with positive BMMNC-Coombs test”). In-depth studies of its pathogenic mechanisms indicated that autoantibodies could inhibit or destroy hemopoietic cells by activating macrophages [8] and complements [9] and blocking functional antigens [10]. The production of autoantibodies in this disease was related to abnormal quantity and functions of B lymphocytes [11], caused by inhibition of regulatory T cells (Treg) [12], T helper (Th) 1, and activated Th2 [13] and Th17 [14] cells. Differentiating IRP from other diseases was beneficial for the treatment of IRP and also other bone marrow failure diseases, such as AA, MDS, and PNH [15, 16]. 

T follicular helper cells (Tfh cells) are a newly defined effector T cell subset that the true helper cells for Ab responses. Tfh cells provide a helper function to B cells and represent one of the most numerous and important subsets of effector T cells. Furthermore, Tfh cells are distinguishable from Th1 and Th2 cells by several criteria, including expressing a unique combination of effector molecules that are critical for their development and function, high levels of the surface receptors ICOS, CD40L (CD154), the cytokine IL-21, the transcription factors Bcl-6, and chemokine receptor CXCR5 [17, 18]. However, aberrant function of Tfh cells could trigger autoimmunity. Dysregulation of Tfh cell function, or expression of Tfh cell-associated molecules such as ICOS or IL-21, contributes to the pathogenesis of certain autoimmune diseases in animal models [19, 20]. However, little is currently known about the potential role of Tfh cells in human autoimmune conditions; there are currently no studies regarding the role of Tfh cells in IRP patients. To understand more specific abnormalities of humoral autoimmunity, we studied the quantity and function of BM Tfh cells in IRP patients and explored the role of Tfh cells in the pathogenesis of this disease.

## 2. Materials and Methods

### 2.1. Patients

All patients were diagnosed as IRP according to the following features [1]: (1) hemocytopenia or pancytopenia with normal or higher percentages of reticulocyte and/or neutrophils; (2) BM: normal or higher percentage of erythroid cells; erythroblastic islands are easy to be seen; (3) exclusion of other primary and secondary hemocytopenia disorders; and (4) BMMNC-Coombs test (+) or/and autoantibodies on the membrane of BM hemopoietic cells (+) tested by flow cytometry (FCM). A total of 90 IRP patients were enrolled in this study. All patients were inpatients of Tianjin Medical University General Hospital from February to December 2010. Forty-three newly diagnosed IRP patients without treatment (14 males, 29 females) were enrolled with a median age of 23 years (range 3–80 years). Forty-seven recovered IRP patients after treatment (20 males, 27 females) were included with a median age of 23 years (range 3–64 years). Twenty-five healthy controls (HC) (9 males, 16 females) from thoracic surgery were also enrolled in this study with a median age of 46 years (range 10–75 years). BM samples were taken from their postoperative discarded ribs. Ethical approval was obtained from Tianjin Medical University, and written informed consent was obtained from all individuals.

### 2.2. BMMNC-Coombs Test

BM mononuclear cells rather than peripheral red cells were used to perform the Coombs test [21]. Fresh heparinized BM samples (5 mL) were diluted with phosphate buffered saline (PBS) in a 1 : 1 proportion, layered over the lymphocyte separation medium, and centrifuged at a low speed for 20 min. Cells were recovered by aspirating the mononuclear cell layer and washing with PBS three times. Cell suspensions of 4-5 × 10^6^/mL in PBS were prepared for testing. Anti-human serum (IgG, IgA, IgM, C3) was diluted as a working solution. Working solutions were mixed with cell suspensions in a 1 : 1 proportion and cultured at 37°C for 30 min. Finally, we observed agglutination by microscopy.

### 2.3. FCM Analysis

Fresh heparinized BM samples (400 *μ*L) were washed with PBS three times, then separated into four tubes, and either stained with mouse IgG1-FITC, mouse IgG1-PE, or mouse IgG1-APC as negative control, or stained separately with CD15-FITC, GlycoA-FITC and CD34-FITC (BD PharMingen, San Diego, CA, USA). Anti-human IgG-PE and anti-human IgM-APC (BD PharMingen) were added to each tube. After incubation in the dark for 30 min at 4°C, cells were incubated with 2 mL erythrocyte lytic solution (BD PharMingen) for 10 min at room temperature and washed three times with PBS. Finally, at least 30 000–100 000 cells were acquired and analyzed on a FACSCalibur flow cytometer (BD Biosciences). 

For phenotypic analysis, fresh heparinized BM samples were stained with anti-human CD4-FITC, CXCR5-APC mAb (R&D, Minneapolis, MN, USA), ICOS-PE, CD40L-PE mAb (BD PharMingen), IL21-PE, CD5-FITC, CD19-PE, IgG-PE, IFN-r, and IL-4 mAb (eBioscience). The staining was performed according to the manufacture's instruction. Finally, the ratios of CD4^+^ CXCR5^+^/CD4^+^, CD4^+^ CXCR5^+^ ICOS^+^/CD4^+^ CXCR5^+^ and CD4^+^ CXCR5^+^ CD40L^+^/CD4^+^ CXCR5^+^, intracytoplasmic CD4^+^ CXCR5^+^ IL-21^+^/CD4^+^ CXCR5^+^, CD19^+^/BMMNC, CD5^+^ CD19^+^/CD19^+^, CD3^+^ CD8^−^IFN-r^+^/CD3^+^ CD8^−^ Lym, CD3^+^ CD8^−^ IL-4^+^/CD3^+^ CD8^−^, and CD3^+^ CD8^−^ IFNr^+^/CD3^+^ CD8^−^ IL-4^+^ cells were analyzed on a FACSCalibur flow cytometer (BD Biosciences).

### 2.4. Western Blot

Hemopoietic cells were separated from BM of patients with IRP and healthy volunteers by erythrocyte lytic solution. Membrane proteins were extracted by Mem-PER Eukaryotic Membrane Protein Extraction Reagent Kit (Thermo Scientific Pierce, Rockford, IL, USA). Protein concentration was determined using BCA Protein Assay Kit (Thermo Scientific Pierce). BM supernatant was stored at −80°C until use. Solubilized proteins were separated using 10% sodium dodecyl sulfate- (SDS-) polyacrylamide gel electrophoresis (PAGE). IgG autoantibodies to BM hemopoietic cells were detected in BM supernatant by western blot [22]. 

### 2.5. Real-Time Polymerase Chain Reaction

For BMMNC Bcl-6 mRNA expression analysis, total RNA was extractedusing Trizol (Invitrogen, Carlsbad, CA, USA), and cDNA was generated using SuperScript III RT kit (Invitrogen, Carlsbad, CA, USA). PCR cycling conditions were initial denaturation at 94°C for 3 minutes followed by 30 cycles at 94°C for 30 seconds, at 58°C for 40 seconds, at 72°C for 1 minute; and 72°C for 5 minutes. *β*-actin served as reference. Primers used are listed as follows: *Bcl-6*: forward 5′-GCC GTG AGC AGT TTA GAG CC-3′, reverse 5′-AGG GAG GTG GCT GTA CAT GG-3′; **β*-Actin*: forward 5′-CAT CAA TGA GCT GCG TGT GGC-3′, reverse 5′-CAG GTC CAG ACG CAG GAT GGC-3′ (synthesized by Shanghai Biotech Co., Ltd., China). Band density of Bcl-6 mRNA was quantified following normalization with *β*-actin. The amplified products were electrophoresed on agarose gel.

### 2.6. Serum Analysis

Total serum IgG and complement levels were measured by rate nephelometry (IMMAGE800 analyzer; Beckman Coulter).

### 2.7. Statistical Analysis

Data were presented as mean ± standard deviation in the text and figures. Statistical differences were considered to be significant at a value of *P* < 0.05. Data were analyzed with GraphPad Prism 5 Software. 

## 3. Results

### 3.1. Detection of Autoantibodies to BM Hemopoietic Cells in IRP Patients

**Figure 1:**
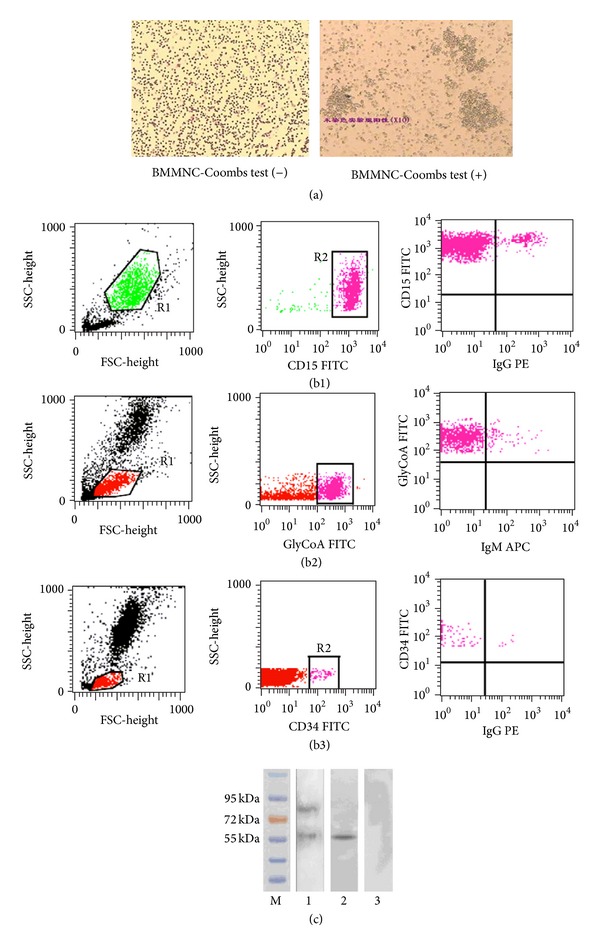
Autoantibodies were detected in IRP patients. (a) BMMNC-Coombs Test. (b) flow cytometry analysis: (b1) autoantibodies were detected on the granulocytes: (b2) autoantibodies were detected on the nucleated erythrocytes. (b3) autoantibodies were detected on the stem cells. (c) The BM cell membrane autoantigens targeted by IgG in IRP were identified by western blot. 1 and 2: IRP patient. 3: healthy control.

We first tried to understand the overall profiles of autoimmunity from 90 patients with IRP. To achieve this, we detected autoantibodies/autoantigens by means of BMMNC-Coombs test, flow cytometry, and western blot using BM samples from 90 patients with IRP and 25 normal controls. The proportion of patients showing positive results in the FCM was 83.3% (75/90), the BMMNC-Coombs test was 70% (63/90), and autoantibody IgG reacting with BM cell membrane antigens could be found in BM supernatant of IRP patients in a positive rate of 60% (54/90), respectively, which were significantly higher than normal controls (0%) (*P* < 0.01) (Figure 1, Table 1).

### 3.2. Increased Frequency of the BM Tfh Cells in IRP Patients

**Figure 2:**
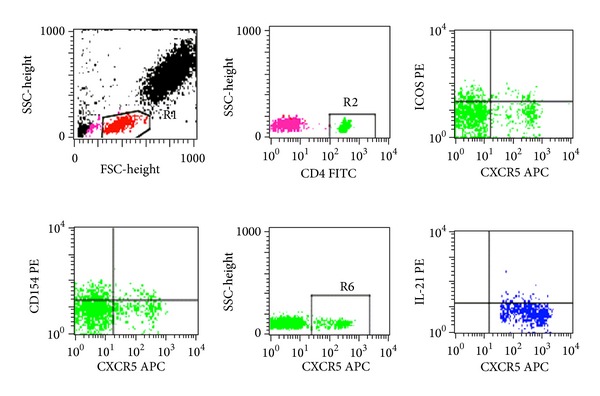
Increased frequency of follicular helper T (Tfh) cells in BM from IRP patients. Representative flow cytometry plots of CD4^+^CXCR5^+^ Tfh cells. Comparison of CD40L (CD154) and ICOS expressions according to the expression of CXCR5 in each CD4 gate. Representative flow cytometry plots of the expression of IL-21 in CD4^+^CXCR5^+^ gate. Percentage of intracytoplasmic CD4^+^CXCR5^+^IL-21^+^ Tfh cells in IRP patients.

**Figure 3:**
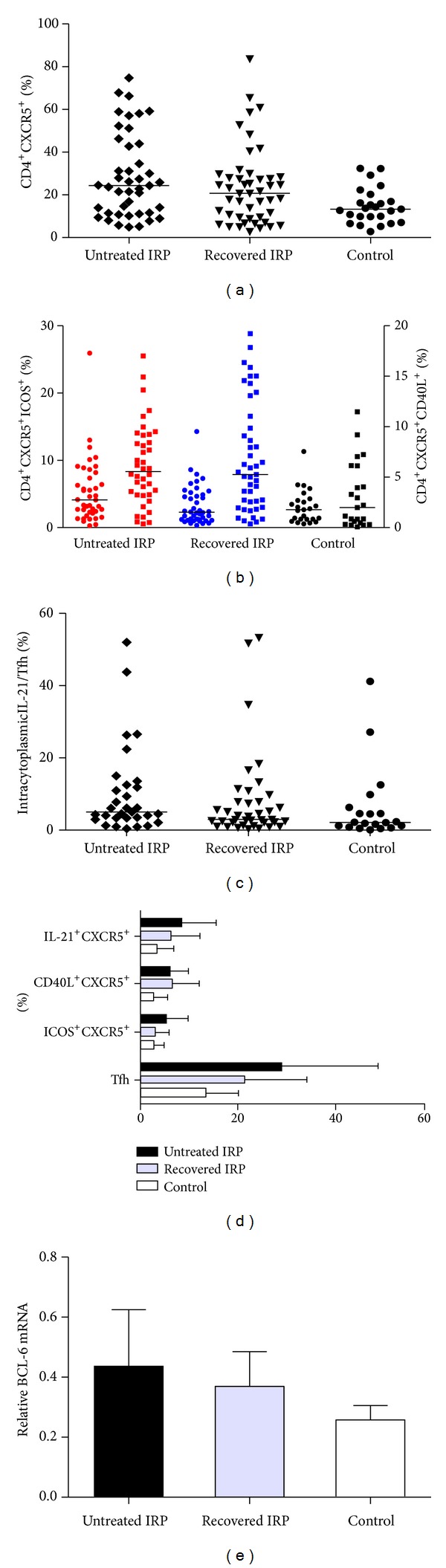
Summary of FACS analysis of BM follicular helper T (Tfh) cells from 90 IRP patients and 25 controls. (a) Tfh cells are expressed as a percentage of CD4^+^ lymphocytes. Each data point represents an individual subject; horizontal lines show the median. Rhombus indicate untreated IRP patients, triangles indicate recovered IRP patients and circles indicate controls. (b) Comparison of ICOS and CD40L expression according to the expression of CXCR5 on CD4^+^ cells in IRP patients and healthy controls. On the left *y*-axis, circles indicate CXCR5^+^ICOS^+^ cells among the CD4 lymphocytes; on the right *y*-axis, squares indicate CXCR5^+^CD40L^+^ cells among the CD4 lymphocytes. Each data point represents an individual subject; horizontal lines show the median. Red indicates untreated IRP patients, blue indicates recovered IRP patients and black indicates controls. (c) Percentage of intracytoplasmic IL-21^+^CXCR5^+^ in CD4 cells in IRP patients, and controls. Each data point represents an individual subject; horizontal lines show the median. Rhombus indicate untreated IRP patients, triangles indicates recovered IRP patients and circles indicate controls. (d) Comparison of Tfh cells, IL-21, ICOS, and CD40L expression, according to the expression of CXCR5 on CD4^+^ cells in IRP patients, and healthy controls. Data correspond to the mean ± SD. (e) Detection of Bcl-6 mRNA expression in IRP patients and healthy controls. Bcl-6 mRNA was estimated by real-time RT-PCR. Data correspond to the mean ± SD.

We analyzed the percentage of CD4^+^CXCR5^+^ Tfh cells in BM from 90 patients with IRP and 25 healthy controls by flow cytometry (Figure 2).  Figure 3(a) showed that the percentage of CD4^+^CXCR5^+^ Tfh cells among CD4^+^ lymphocytes in untreated IRP patients was significantly higher than that in recovered IRP patients and healthy controls and that of recovered IRP patients was higher than that in healthy controls (28.79 ± 19.70% versus 21.15 ± 12.81% versus 13.42 ± 6.72%, *P* < 0.05).

### 3.3. Increased Percentage of ICOS, CD40L, and IL-21 Levels on BM Tfh Cells and Bcl-6 mRNA Expression in IRP Patients

There were increased proportion of CD4^+^CXCR5^+^ICOS^+^ Tfh cells of untreated IRP patients compared with healthy controls (5.04 ± 4.71% versus 2.96 ± 2.89% versus 2.99 ± 2.23%, *P* < 0.05). There were increased proportion of CD4^+^CXCR5^+^CD40L^+^ Tfh cells of untreated IRP patients compared with healthy controls (5.87 ± 4.14% versus 6.52 ± 5.47% versus 2.93 ± 2.92%, *P* < 0.05) (Figure 3(b)). The fraction of intracytoplasm CD4^+^ CXCR5^+^ IL-21^+^ in Tfh cells of untreated and recovered IRP patients were significiantly higher than that of controls (8.20 ± 7.41% versus 6.30 ± 6.03% versus 3.43 ± 3.40%, *P* < 0.05) (Figure 3(c)). We assessed the expression of transcription factor Bcl-6 in IRP patients and healthy controls by real-time polymerase chain reaction. Our data showed that the relative expression of Bcl-6 mRNA in BMMNC of untreated IRP patients was significiantly higher than that of recovered IRP patients and controls. That in recovered IRP patients was higher than that in controls (0.625 ± 0.248 versus 0.485 ± 0.253 versus 0.306 ± 0.210, *P* < 0.05) (Figure 3(e)).

### 3.4. The Frequency of Tfh Cells and the Expression of Tfh-Related Molecules Were Associated Closely with Clinical Characteristics in IRP Patients

To determine whether the BM Tfh cell phenotype might identify a pathogenically distinct subset of patients with IRP or whether it simply reflects disease activity, we compared clinical and BM evidence of disease activity at the time the BM Tfh cell phenotype was initially studied. In IRP group, there were positive correlations between the frequency of BM CD4^+^CXCR5^+^ Tfh cells and the expression of Bcl-6 mRNA, the ratio of CD19^+^/BMMNC, the ratio of CD3^+^CD8^−^IL-4^+^/CD3^+^CD8^−^, the ratio of CD34^+^IgG^+^/BMMNC, and the level of serum IgG (*r* = 0.474, 0.252, 0.475, 0.314, 0.376, resp., *P* < 0.05); there were negative correlations between the frequencies of BM.

**Figure 4:**
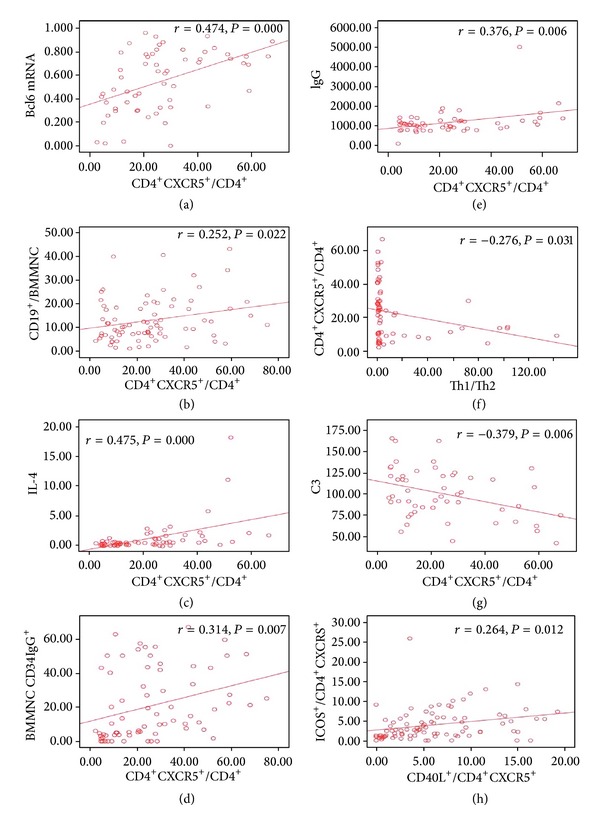
Correlation of Tfh cells frequency in BM and clinical evidence in IRP patients. (a) Relationship between the frequency of the BM CD4^+^CXCR5^+^ Tfh cells and the expression of Bcl-6 mRNA in IRP group. (b) Relationship between the frequency of the BM CD4^+^CXCR5^+^ Tfh cells and the ratio of CD19^+^/BMMNC in IRP group. (c) Relationship between the frequency of the BM CD4^+^CXCR5^+^ Tfh cells and the ratio of CD3^+^CD8^−^IL-4^+^/CD3^+^CD8^−^ in IRP group. (d) Relationship between the frequency of the BM CD4^+^CXCR5^+^ Tfh cells and the ratio of CD34^+^IgG^+^/BMMNC in IRP group. (e) Relationship between the frequency of the BM CD4^+^CXCR5^+^ Tfh cells and the total serum IgG level in IRP group. (f) Relationship between the frequency of the BM CD4^+^CXCR5^+^ Tfh cells and the ratio of Th1/Th2 in IRP group. (g) Relationship between the frequency of the BM CD4^+^CXCR5^+^ Tfh cells and the level of serum C3 in IRP group. (h) Scatterplot of the percentage of CD4^+^CXCR5^+^ICOS^+^ cells versus CD4^+^CXCR5^+^CD40L^+^ cells from 90 IRP patients showing a correlation.

**Figure 5:**
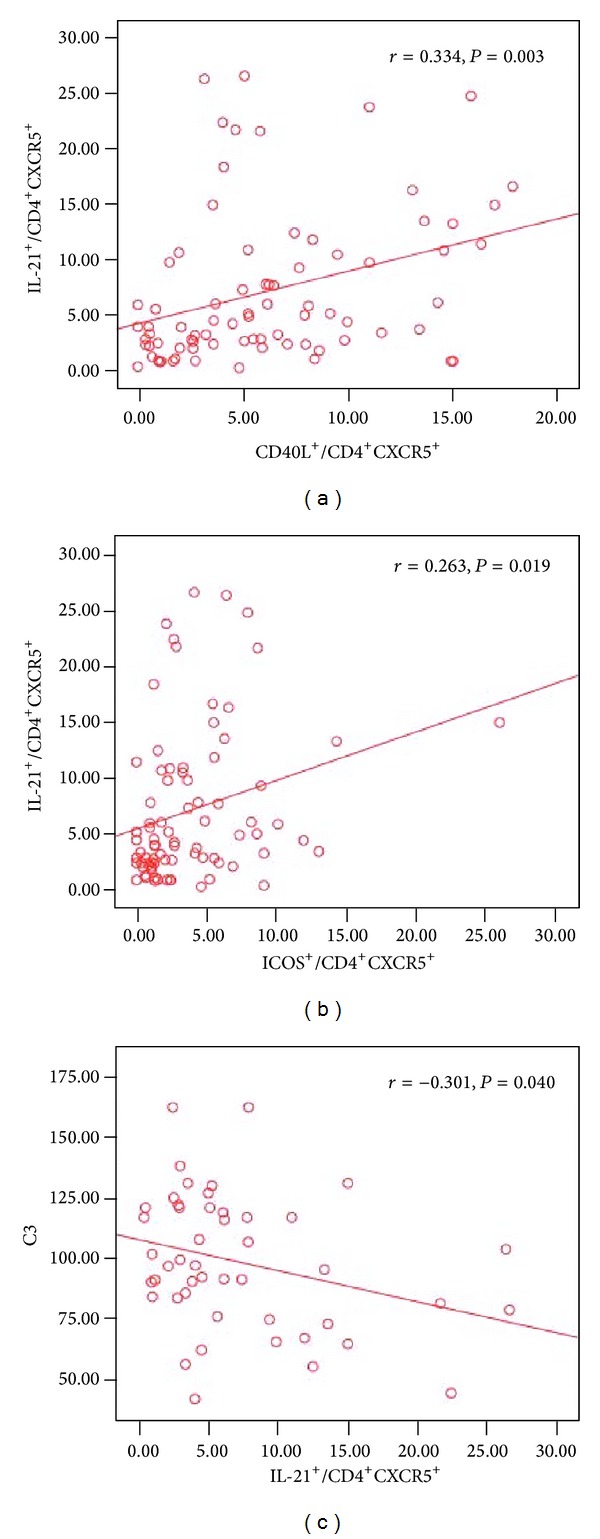
Correlation of the ratio of CD4^+^CXCR5^+^IL-21^+^ cells with the frequency of Tfh cells in BM in IRP patients. (a) Relationship between the ratio of CD4^+^CXCR5^+^IL-21^+^ cells and the ratio of CD4^+^CXCR5^+^CD40L^+^ cells. (b) Relationship between the ratio of CD4^+^CXCR5^+^IL-21^+^ cells and the ratio of CD4^+^CXCR5^+^ICOS^+^ cells. (c) Relationship between the ratio of CD4^+^CXCR5^+^IL-21^+^ cells and the level of serum C3 in IRP group.

CD4^+^CXCR5^+^ Tfh cells and the ratio of Th1/Th2, the level of serum C3 (*r* = −0.276, −0.379, resp., *P* < 0.05). There was positive correlation between the ratios of CD4^+^CXCR5^+^CD40L^+^ and CD4^+^CXCR5^+^ICOS^+^ (*r* = 0.264, *P* < 0.05) (Figure 4). There were positive correlations between the ratios of CD4^+^CXCR5^+^IL-21^+^ and CD4^+^CXCR5^+^CD40L^+^, CD4^+^CXCR5^+^ICOS^+^, the expression of Bcl-6 mRNA (*r* = 0.334, 0.263, 0.332, resp., *P* < 0.05), there was negative correlation between the ratio of CD4^+^ CXCR5^+^ IL-21^+^ and the level of serum C3 (*r* = −0.301, *P* < 0.05) (Figure 5).

## 4. Discussion

This study is the first to describe an alteration of BM Tfh cells of patients with IRP. IRP is a disease differentiated from other bone marrow failure diseases, which have been described in recent years [2, 3, 15]. Coincidentally, some scholars have also detected the presence of autoantibodies in some acquired AA patients. The study indicated that haematopoietic cells are the targets of immune abnormality in some bone marrow failures (BMF). The autoantibodies expressed in BMF may be powerful clinical parameter to distinguish patients associated with immune abnormality from bone marrow failure syndrome patients [23]. We also found that there existed autoantibodies on BM hemopoietic cells when using BMMNC-Coombs test and/or flow cytometry in these patients. An in-depth study of its pathogenic mechanism showed that IRP is an autoimmune BM failure disease where autoantibodies target BM hemopoietic cells. The BM failure of this disease is mediated by autoantibodies on the membrane of BM haemopoietic cells, which can inhibit haemopoiesis and thereby induce hemocytopenia. A study by Liu et al. [22] showed that the 30 kDa protein band as autoantigen was successfully identified in IRP. Furthermore, we found that IRP was an autoimmune disease characterized by alterations of B-cell and T-cell subsets, Treg cells depletion, and an increase in Th2 and Th17 cells [11–14].

It is now apparent that a subset of nonpolarized CD4^+^ T cells, termed Tfh cells, are the true helper cells for Ab responses. Tfh cells provide a helper function to B cells and represent one of the most numerous and important subsets of effector T cells [17]. Furthermore, Tfh cells express a unique combination of effector molecules that are critical for their development and function, including high levels of the surface receptors ICOS, CD40L, the cytokine IL-21, and the transcription factors Bcl-6. These molecules play critical roles in promoting the activation, differentiation, and survival of B cells and/or CD4^+^ T cells [24]. Although Tfh cells are critical for the generation of effective long-lived protective Ab responses, dysregulation of Tfh cell function or expression of Tfh cell-associated molecules could contribute to the pathogenesis of certain autoimmune diseases. This has indeed been established in both animal models and humans with autoimmune diseases, particularly in conditions associated with autoantibodies. 

The first suggestion that Tfh cells could underlie immune-mediated diseases was their overabundance in several murine models of SLE [25] and the fact that preventing Tfh formation reduced disease [26]. Recent studies indicate that excessive generation of Tfh cells likely contributes to the production of pathogenic auto-Abs in several human autoimmune conditions. Circulating Tfh-like cells are also found at increased frequencies in the blood of some patients with SLE, Sjogren's syndrome [27], juvenile dermatomyositis [28], RA [29], and AITD [30], which are autoimmune conditions characterized by the production of pathogenic auto-Abs. Importantly, the expansion of Tfh-like cells in SLE correlated with auto-Abs titers and tissue damage.

Zaidan et al. [31] reported on a patient of IgG4-related systemic disease with tubulointerstitial nephritis; striking features were the abundance of interfollicular plasma cells and CD4^+^ T-cells in germinal centres of lymph nodes and the dramatic response to rituximab point to possible roles of Tfh cells in enhancing a skewed B-cell terminal maturation and of CD20^+^ B cells in disease progression.

CXCR5 was an early defining marker of Tfh cells. Expression of CXCR5 allows Tfh cells to relocate from the T-cell zone to the B cell follicles, where they are positioned to directly support B cell expansion and differentiation. Importantly, CD4^+^CXCR5^hi^ T-cells are capable of supporting Ig class switching and Ab secretion by naive, memory, and GC B cells in vitro [32]. Effector CD4^+^ T-cells deliver help to antigen-specific B-cells in an MHC class II-restricted manner. In the absence of signaling through ICOS/ICOS-L or CD40/CD40L, CD4^+^ T cells fail to upregulate CXCR5, resulting in reduced numbers of Tfh cells and impaired GC formation [33]. This is consistent with reduced frequencies of circulating CD4^+^CXCR5^+^ T cells in patients with mutations in ICOS or CD40L [34]. IL21-producing CD4^+^ T cells are central to humoral immunity. IL-21 is a cytokine with pleiotropic actions, promoting terminal differentiation of B cells, increased immunoglobulin (Ig) production, and the development of Th17 and Tfh cells. IL-21 is also implicated in the development of autoimmune disease and has antitumor activity. Reminiscent of Tfh cells, they express Bcl-6 and CXCR5 and provide help to B-cells via the production of IL-21; increased IL-21 contributed to the enhanced B-cell help functions of Tfh cells [35, 36]. For Tfh cells, a similar “master regulator” function has now been ascribed to Bcl-6, and several studies have now shown that Bcl-6 deficiency inhibits, and overexpression promotes, the generation of Tfh cells [37–40]. These findings firmly established a critical role for Bcl-6 in coordinating Tfh formation.

In this study, we have investigated the quantity and function of BM Tfh cells in IRP patients. The results demonstrated that the frequency of CD4^+^CXCR5^+^ Tfh cells, CD4^+^CXCR5^+^ICOS^+^, CD4^+^CXCR5^+^CD40L^+^, and intracytoplasmic CD4^+^ CXCR5^+^ IL-21^+^ on Tfh cells and the expression of Bcl-6 mRNA in BMMNC were markedly expanded in IRP subjects as compared with healthy controls. In conclusion, we hypothesized that IRP is a bone marrow abnormality different from other known hemopoietic diseases and this autoimmune disease is characterized by BM failure which maybe mediated by autoantibodies on the membrane of BM haemopoietic cells. Our findings indicated that there existed increased frequency and hyperfunction of BM Tfh cells in IRP patients which were positively correlated with disease progression, including the presence of autoantibodies, disease activity, and the response to treatment. Thus, it might be possible that dysregulated Tfh cells were involved in the pathogenesis of IRP and antagonizing Tfh cells and the molecules they express could be a novel strategy for inhibiting the disease progression. However, this study points to some aspects that raise hypotheses on its pathogenesis; further investigations are required to provide a thorough understanding of the complex mechanisms of the disease, especially involved the B cells and Th subsets, which might lead to disrupt the cytokine network as a further means of inhibiting the initiation and/or progression of IRP.

## Figures and Tables

**Table 1:** Detection of autoantibodies to BM hemopoietic cells in IRP patients.

	IgG	IgM	C3	IgA
BMMNC-Coombs test	(41/90) 45.6%	(27/90) 30%	(23/90) 25.6%	(8/90) 8.9%
FCM analysis	(59/90) 65.6%	(66/90) 73.3%	—	—
Western blot	(54/90) 60%	—	—	—

BMMNC: bone marrow mononuclear cell; FCM: flow cytometry; Ig: immunoglobulin.
